# AbaComplex Enhances Mitochondrial Biogenesis and Adipose Tissue Browning: Implications for Obesity and Glucose Regulation

**DOI:** 10.3390/foods14010048

**Published:** 2024-12-27

**Authors:** Serena Sagliocchi, Elisabetta Schiano, Lucia Acampora, Fortuna Iannuzzo, Annunziata Gaetana Cicatiello, Caterina Miro, Annarita Nappi, Federica Restolfer, Mariano Stornaiuolo, Stefano Zarrilli, Fabrizia Guerra, Gian Carlo Tenore, Monica Dentice, Ettore Novellino

**Affiliations:** 1 Department of Clinical Medicine and Surgery, University of Naples Federico II, 80131 Napoli, Italy; serena.sagliocchi@unina.it (S.S.); lucia.acampora@unina.it (L.A.); annunziatagaetana.cicatiello2@unina.it (A.G.C.); caterina.miro@unina.it (C.M.); annarita.nappi@unina.it (A.N.); f.restolfer@gmail.com (F.R.); stefano.zarrilli@unina.it (S.Z.); 2 Inventia Biotech-Healthcare Food Research Center s.r.l., Strada Statale Sannitica KM 20.700, 81020 Caserta, Italy; elisabettaschiano@inventiabiotech.com (E.S.); fabrizia.guerra@inventiabiotech.com (F.G.); ettorenovellino@inventiabiotech.com (E.N.); 3 Department of Pharmacy, University of Chieti-Pescara G. D’Annunzio, 66100 Chieti, Italy; fortuna.iannuzzo@unich.it; 4 Department of Pharmacy, University of Naples Federico II, Via Domenico Montesano 59, 80131 Napoli, Italy; mariano.stornaiuolo@unina.it (M.S.); giancarlo.tenore@unina.it (G.C.T.); 5 Faculty of Medicine and Surgery, Catholic University of the Sacred Heart, 00168 Rome, Italy

**Keywords:** abscisic acid, nutraceutical, browning, thermogenesis, obesity, glycemic control

## Abstract

Adipose tissue, particularly white adipose tissue (WAT), plays a central role in energy storage and metabolic regulation. Excess WAT, especially visceral fat, is strongly linked to metabolic disorders such as obesity and type 2 diabetes. The browning of WAT, whereby white fat cells acquire characteristics of brown adipose tissue (BAT) with enhanced thermogenic capacity, represents a promising strategy to enhance metabolic health. In this study, we investigated the effects of chronic supplementation with an infusion based on lyophilized, thin nectarines rich in abscisic acid (ABA), named AbaComplex, on promoting browning of WAT and activating BAT in mice. Over 30 days, C57BL/6 mice were treated with the ABA-rich infusion, and various metabolic and molecular parameters were assessed. The results showed that the AbaComplex significantly increased the expression of browning markers, such as UCP1 and PGC1-α, in both visceral and subcutaneous WAT. Additionally, mitochondrial biogenesis and function were enhanced, evidenced by elevated mitochondrial DNA content and activity. The treatment also reduced the weight of WAT (both visceral and subcutaneous) and BAT and significantly improved glucose uptake in WAT via upregulation of GLUT4, suggesting enhanced insulin sensitivity. Overall, the pronounced browning effect in WAT underscores the potential of AbaComplex as a natural approach for combating obesity and improving metabolic health.

## 1. Introduction

Abscisic acid (ABA) is a plant hormone recognized for its role in responding to environmental stress and regulating seed dormancy and germination in plants [1]. Interestingly, recent studies have identified ABA as an endogenous hormone in mammals, with significant implications for glucose metabolism and immune function [2,3]. This hormone, through its receptor lanthionine synthetase C–like protein 2 (LANCL2) [4], stimulates glucose uptake and enhances glycemic control, suggesting a potential role in the regulation of metabolic processes [5]. Adipose tissue (AT), including white adipose tissue (WAT) and brown adipose tissue (BAT), plays an essential role in energy balance and glucose homeostasis [6]. While WAT primarily functions in energy storage, BAT is specialized in energy expenditure through thermogenesis [7]. The ability of BAT to burn fatty acids and generate heat positions it as a key player in combating obesity and metabolic disorders [8]. Moreover, recent discoveries have shown the presence of brown fat-like cells, or beige cells, within WAT, capable of enhancing whole-body energy expenditure. These beige cells share characteristics with classical brown adipocytes, including high mitochondrial content and uncoupling protein 1 (UCP1) expression, which is essential for thermogenesis [9]. Among the key transcriptional factors influencing the development of brown and white adipocytes, the peroxisome proliferator-activated receptor gamma (PPARγ) is recognized as the main regulator of adipocyte differentiation [10]. The interaction between PPARγ and PR domain-containing protein 16 (PRDM16), along with the PPARγ coactivator 1α (PGC1α), triggers the specific activation of brown adipogenic and thermogenic gene expression [11]. Additionally, PPARγ stimulates PGC1-α, which in turn enhances PPARγ activity through positive feedback and improves the expression of UCP1 [12,13], a crucial component in the controlled uncoupling of mitochondrial oxidative phosphorylation. Based on previous findings that ABA promotes the translocation of glucose transporters-4 (GLUT4) to the plasma membrane in mouse pre-adipocytes, a study published by Sturla and colleagues explored the role of the ABA/LANCL2 system in regulating glucose uptake and metabolism in 3T3-L1 adipocytes [14]. Compared with insulin, ABA treatment of adipocytes resulted in lower CO_2_ production, triglyceride accumulation, and synthesis of glucose-derived fatty acids. Although ABA did not induce differentiation of pre-adipocytes in vitro, it was able to stimulate remodeling in fully differentiated cells, leading to a reduction in cell size, an increase in the number of mitochondria, enhanced O_2_ consumption, and improved transcription of BAT genes [14]. More specifically, synthetic ABA was demonstrated to significantly upregulate the expression of browning-specific markers such as UCP1 and PGC1-α. Furthermore, in vivo experiments with CD1 mice have shown that chronic ABA administration with a single dose of oral ABA (1 μg/kg body weight) led to upregulation of browning genes and mitochondrial content in WAT, compared with untreated controls.

Interestingly, thinned nectarines (TN), which are typically an agro-food waste product, have emerged as a rich source of ABA [15]. In fact, during the cultivation of immature fruits, a substantial portion of these latter is usually discarded to allow the remaining fruits to grow to a marketable size [16]. These discarded immature fruits, however, are particularly rich in ABA, as this compound is not only recognized for its ability to regulate glycemic control in humans but also represents a phytohormone with a well-documented role in the regulation of plant growth [17]. Following a comprehensive screening of various fruit matrices, including apples, pears, plums, and apricots, TN were identified as having the highest concentration of ABA [18]. This finding highlights the potential of TN as a high-value source of ABA compared with other commonly discarded fruits, making them particularly attractive for beneficial applications on health outcomes. Indeed, by repurposing these otherwise discarded fruits, the agricultural industry can not only reduce waste but also create valuable health-promoting products [19]. The presence of other bioactive compounds in TNs, such as polyphenols, further enhances their antioxidant and antidiabetic properties, contributing to their overall efficacy as a nutraceutical [3,15]. In this regard, a recent study has demonstrated that a nutraceutical formulation based on TNs significantly improved glycemic homeostasis, highlighting their potential role in managing glucose homeostasis. In particular, a randomized controlled trial (RCT) conducted on patients with type 2 diabetes mellitus (T2DM) showed that TN-based treatments led to a significant reduction in fasting plasma insulin and overall glycemic levels, with a notable correlation between ABA plasma levels and glycemia [3]. Based on these observations and given the emerging evidence of ABA’s involvement in glucose metabolism and adipose tissue function, this study aims to investigate the effects of AbaComplex infusion, rich in abscisic acid, on the browning process in mice. The primary objective is to determine whether this latter can stimulate BAT activity and enhance the browning of white fat, thus potentially offering a novel approach to managing obesity and related metabolic diseases. The significance of this study lies in its potential to uncover a natural strategy to promote fat browning and enhance metabolic health, based on the hypothesis that the AbaComplex infusion can increase BAT activity and induce browning in WAT, leading to improved glucose disposal and management in mice.

## 2. Materials and Methods

### 2.1. Mouse Strains

Male C57BL/6J mice, aged 12 weeks, were obtained from Jackson Laboratory (Bar Harbor, ME, USA) and utilized as littermates in this study. The animals were treated following both national and European community guidelines, with the experimental protocols receiving approval from the Animal Research Committee of the University of Naples “Federico II”. The mice were kept in cages with fresh bedding under the following controlled environmental conditions: a temperature range of 20–24 °C, relative humidity between 50–60%, and a light–dark cycle of 14:10 h. All animals had unrestricted access to food and water throughout the study. Animal procedures received approval from the Institutional Animal Care and Use Committee (IACUC) (protocol no. 354/2019-PR).

### 2.2. ABA Dietary Supplementation and Denervation Experiments

C57BL/6 mice, both male and female, aged 12 weeks (sources from The Jackson Laboratory, Bar Harbor, ME, USA), were randomly assigned into four groups. These included the male-CTR (*n* = 7) and female-CTR (*n* = 7) groups, which were provided with a standard diet, and the male-ABA group (*n* = 7) and female-ABA group (*n* = 7), which received dietary supplementation for 30 days (chronic treatment) before the sacrifice. Daily food and water intake was assessed in individually housed mice. Body weight was recorded twice a week. Subcutaneous and visceral fat and gastrocnemius and tibial muscles were weighed. For the ABA treatment, an infusion was prepared using tea bags containing lyophilized, thin nectarines named AbaComplex (ABA-C), providing an ABA dose of 12 µg/kg of mouse body weight (BW)/day. Moreover, to assess the stability of ABA in the ABA-C-based infusion, HPLC-DAD quantification was carried out at intervals of 1, 4, 7, 21, and 28 days. Mice from each experimental group were collected for further testing.

### 2.3. HPLC Analysis of ABA Content

The ABA content in the infusion was analyzed using a Jasco Extrema LC-4000 HPLC system (Jasco Inc., Easton, MD, USA) equipped with an autosampler, a binary solvent pump, a diode-array detector (DAD), and a fluorescence detector (FLD). The separation was carried out on a Kinetex^®^ C18 column (250 mm × 4.6 mm, 5 μm; Phenomenex, Torrance, CA, USA). The mobile phases used were water at 1% formic acid in water (A) and 1% formic acid in acetonitrile (B). After an initial hold at 5% solvent B for 3 min, elution conditions were as follows: a gradient from 5% (B) to 75% (B) over 20 min, followed by a 1 min hold; subsequently, the column was re-equilibrated to starting conditions for another minute. The separation parameters included a column temperature of 30 °C, an inject volume of 20 µL, and a flow rate of 1 mL/min. ABA was detected at 265 nm, with peak identifications based on retention times and standard addition to samples. Quantification was achieved using a calibration curve made with six different concentrations, ranging from 0.1 to 100 ppm, with each concentration measured in triplicate.

### 2.4. Real-Time qRT-PCR

Total DNA was extracted from tissue samples using established methods. For analyzing mtDNA content, real-time PCR with SYBR Green was conducted using primers specific to a region in the mouse mtDNA ND1 gene and primers specific to RNaseP, a single-copy nuclear reference gene, as previously outlined [20] (Table S1). The relative copy number was derived from threshold cycle value (DCt = nucDNA Ct-mtDNA Ct), and the mtDNA copy number per cell was calculated as 2 × 2DCt, considering the two copies of RNaseP present in each nucleus [21].

For transcript analysis, total RNA was isolated from muscle samples frozen in liquid nitrogen using Trizol, following the manufacturer instructions (Invitrogen). Complementary DNAs (cDNAs) were synthesized with Vilo reverse transcriptase (Life Technologies) as indicated by the manufacturer. The cDNAs were amplified via PCR in an iQ5 Multicolor real-time Detector System (BioRad) with the fluorescent double-stranded DNA-binding dye SYBR Green (BioRad). Primers specific to each gene were optimized to work under uniform cycling conditions (95 °C for 10 min, followed by 40 cycles at 95 °C for 15 s and 60 °C for 1 min), producing amplicons of approximately 200 bp for each amplification. Whenever feasible, primers spanned an exon–exon junction, and RNA was digested with DNAse to avoid genomic DNA interference. Primer sequences are listed in Table S1 Cyclophilin A served as the internal control to calculate relative gene expression levels. All samples were tested in triplicate, and results, expressed as N-fold differences in target gene expression, were calculated using the formula 2 − (ΔCt target − ΔCt control) (Livak and Schmittgen, 2001).

### 2.5. Mitochondrial Staining

Mitochondrial activity was assessed using the MitoTracker™ Red CMXRos dye (Invitrogen) as per the manufacturer’s protocol. At first, adipose tissue was cut into pieces of 3–4 mm using sterile scissors and then washed multiple times in Dulbecco’s modified Eagle’s medium (DMEM, HiMedia Leading BioSciences Company, Mumbai, Maharashtra, India, product no: AL007-500ML) supplemented with 1% penicillin/streptomycin (Gibco, Thermo Fisher Scientific, Waltham, MA, USA, product number: 15070063). Following the manufacturer’s guidelines, the tissue was digested with collagenase type IV (100 U/mL, Gibco, Thermo Fisher Scientific, Waltham, MA, USA, catalog number: 17104-019). After 1 h of digestion, the enzyme was neutralized by adding two volumes of DMEM + 10% fetal bovine serum (FBS, HiMedia Leading BioSciences Company, Mumbai, Maharashtra, India, product number: RM10432). Mature adipocytes were then isolated by centrifugation at 1200× *g* for 10 min to form a pellet. The cells were incubated in pre-warmed PBS containing 50 nM MitoTracker TMRE at 37 °C for 30 min in the dark [22]. Prior to analysis, the cells were washed twice and subsequently analyzed using the FACS Canto2 flow cytometer (Becton Dickinson, Franklin Lakes, NJ, USA).

### 2.6. Histology

Formalin-fixed tissues, including white and brown adipose tissue, were embedded in paraffin and sectioned to a thickness of 7 μm. These sections were stained with hematoxylin and eosin (HE) following standard protocols (Dubowitz, 1974). In brief, sections were fixed in 4% formaldehyde for 15 min at room temperature and subsequently stained with HE.

### 2.7. Metabolic Parameters Measurements

Serum from each blood sample was obtained by centrifugation at 3000 rpm for 20 min. Serum total cholesterol, low-density lipoprotein (LDL) cholesterol, high-density lipoprotein (HDL) cholesterol, triglyceride levels were evaluated using commercially available biochemical assay kits (Bionova s.r.l., Naples, Italy). To evaluate the glycemic response following glucose load, a glucose tolerance test (GTT) was conducted at the end of the chronic treatment, after an overnight fast. Blood glucose levels were measured at baseline (0 min) and at 30, 60, 90, and 120 min following an intraperitoneal injection of beta D-glucose [23] (2 mg/g body weight; Sigma-Aldrich, St. Louis, MO, USA). Additionally, an acute GTT was conducted on the same groups of mice 15 min after oral gavage administration of an ABA-C extract, solubilized with a DMSO–NaCl buffer to deliver the total daily ABA dose used in the chronic experiments. Similar to the chronic GTT, blood glucose levels were measured at baseline (0 min) and at 30, 60, 90, and 120 min post intraperitoneal injection of beta D-glucose.

### 2.8. Quantification and Statistical Analysis

Results are presented as the mean ± standard deviation (SD) throughout, as detailed in the figure legends. Multiple group comparisons were evaluated using a two-way ANOVA test followed by Bonferroni post-hoc analysis. Relative mRNA levels, with the control sample set arbitrarily at 1, are shown as outcomes of real-time qRT-PCR, using Cyclophilin A as the reference housekeeping gene. In all experiments, statistical significance was defined as a *p*-value less than 0.05. Asterisks indicate significance levels as follows: * *p* < 0.05, ** *p* < 0.01, and *** *p* < 0.001. Definitions of the *n*-values are specified in the figure legends.

## 3. Results

### 3.1. ABA-Extract Characterization and Treatment

HPLC analysis demonstrated that the infusion prepared using 2 g of AbaComplex (ABA-C) dissolved in 100 mL of water contained an amount of 20 µg of ABA. Considering that a mouse typically consumes approximately 2.5 mL of water per day, the infusion prepared by dissolving 2 g of ABA-C in 100 mL of water contained about 0.5 µg of ABA, meeting the aforementioned daily dose per kg of BW. Moreover, stability tests performed from day 1 to day 28 revealed that the ABA concentration remained constant throughout the indicated time period. This stability suggests that the infusion maintains its ABA concentration consistently, ensuring the efficacy of the treatment throughout the studied storage period. Despite the excellent stability of ABA in the infusion, it was freshly prepared twice a week for the in vivo experiments and stored in the refrigerator.

### 3.2. Mitochondrial Function Is Improved by Chronic Treatment with ABA-C

**Figure 1:**
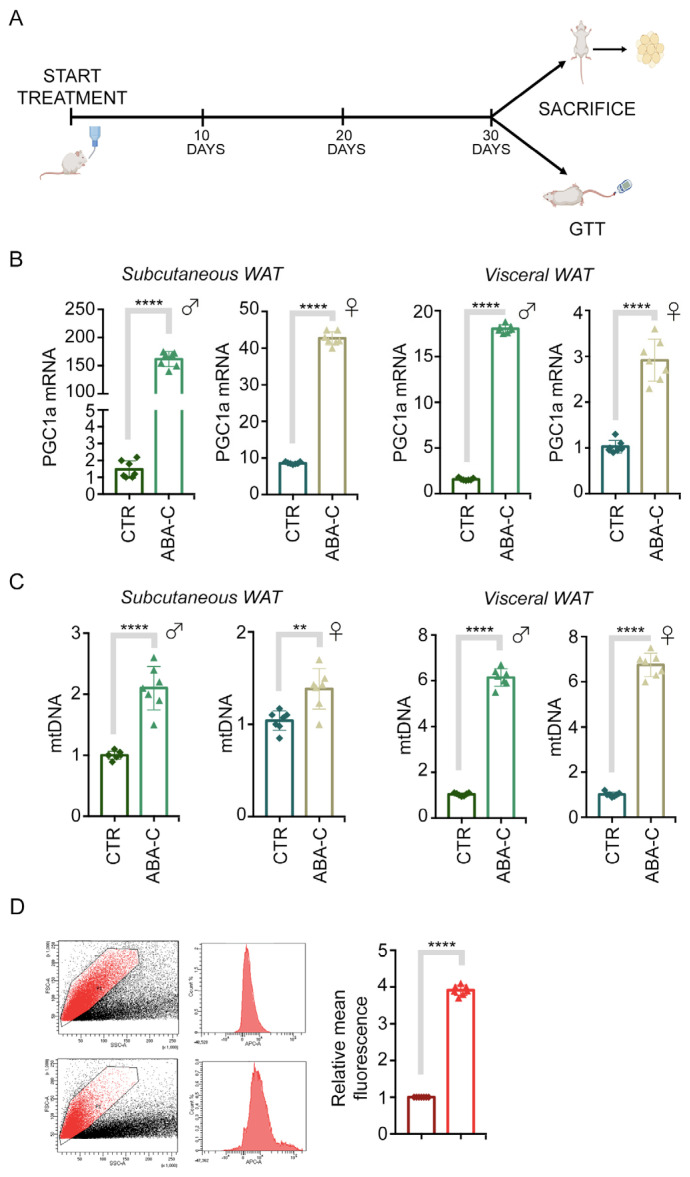
Chronic ABA-C treatment promotes mitochondrial function in WAT. (**A**) Schematic representation of the experimental plan. C57/Bl6 wild-type mice were treated or not treated with ABA-C for 30 days, and then one group of animals was sacrificed, and visceral and subcutaneous WAT were collected while another group of animals was used for glycemic testing. (**B**) mRNA expression analysis of PGC1-α in the visceral and subcutaneous WAT of ABA-C-treated animals compared with controls, in both males and females. Cyclophilin A was used as an internal control. Normalized copies of the indicated genes in CTR were set as 1. (**C**) Measurement of mitochondrial DNA copy number (mtDNA-CN) by real-time PCR in the visceral and subcutaneous WAT of ABA-C-treated animals compared with controls, in both males and females. (**D**) Evaluation of mitochondrial activity by FACS analysis in pre-adipocytes obtained from the subcutaneous WAT of control versus ABA-C-treated mice. Data in all graphs are shown as mean ± SD from three separate experiments. Statistical analyses were performed using the *t*-test analysis. *n* = 7. ** *p* < 0.01; **** *p* < 0.0001.

We tested whether ABA-C preserves the beneficial effects of pure ABA on adipose tissue, stimulating mitochondrial biogenesis, glucose uptake and respiratory uncoupling, and expression of browning genes in white adipocytes of chronically ABA-C-treated mice. To assess the potential effects on ABA-C-induced alterations in mitochondrial content and function, mice were given a standard chow diet with or without ABA-C administered via drinking water. After 30 days, the animals were sacrificed, and both visceral and subcutaneous WAT samples were collected to determine the mitochondrial profile (Figure 1A). First, we measured the expression level of the master regulator of mitochondrial biogenesis and browning of white adipocytes, PGC1-α, and found a significant induction of PGC1-α in the WAT of ABA-C-treated animals compared with controls, in both males and females (Figure 1B). In addition, WAT from the ABA-C-treated group with higher PGC1α expression had a significantly higher mitochondrial DNA copy number (mtDNA-CN), in both males and females, as assessed by quantitative real-time PCR (Figure 1C). Thus, mtDNA-CN copy number had a significant positive correlation with PGC1-α expression. Consistently, a similar increase was detected in mitochondrial activity when pre-adipocytes derived from subcutaneous WAT of control or ABA-C-treated mice were assessed using MitoTracker™ Red CMXRos (MTR), showing an increase in mitochondrial activity in subcutaneous WAT from the ABA-C-treated group compared with the control group (Figure 1D). Taken together, these results indicate that oral ABA-C, taken for 30 days, significantly increased mitochondrial DNA and activity in WAT from ABA-C-treated as compared with control mice, as well as exposure to ABA-C increased the capacity for mitochondrial biogenesis stimulating PGC1-α expression in WAT from treated mice.

### 3.3. Chronic ABA-C Treatment Induces Browning of Subcutaneous WAT

**Figure 2:**
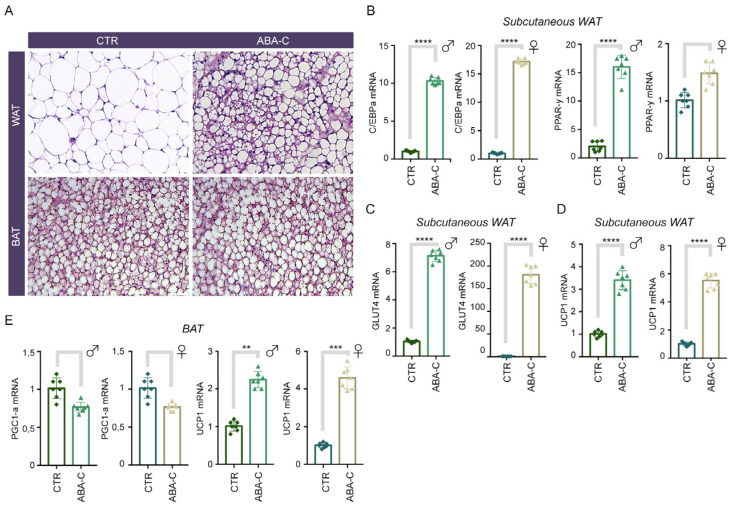
Browning of subcutaneous WAT was induced by chronic ABA-C treatment. (**A**) Hematoxylin & eosin (H&E) staining was performed on WAT sections of ABA-C-treated and control (CTR) mice. Magnification 20×, scale bar 50 μm. (**B**–**D**) mRNA expression analysis of C/EBP-αEBPαa and PPAR-gγ, GLUT4, and UCP1 in subcutaneous WAT of ABA-C-treated animals compared with controls, in both males and females. (**E**) mRNA expression analysis of PGC1-α a and UCP1 in BAT of ABA-C-treated animals compared with controls, in both males and females. Cyclophilin A was used as the internal control. Normalized copies of the indicated genes in CTR were set as 1. The data represent the means ± SD from three separate experiments. *n* = 7. ** *p* < 0.01; *** *p* < 0.001; **** *p* < 0.0001.

Then, we investigated the possible browning effect of ABA chronic treatment on adipose tissue in vivo. Following 30 days of ABA-C treatment, we observed that, from a histological perspective, ABA-C treatment only induced browning of subcutaneous WAT compared with untreated controls, but no change in BAT (Figure 2A). To this end, we measured the expression of master regulators of adipogenesis, C/BPE-α and PPAR-γ, in subcutaneous WAT from ABA-C-treated and control groups. All markers examined were significantly upregulated in subcutaneous WAT from ABA-C-treated animals compared with controls, both in males and females (Figure 2B). In this line, we also investigated whether ABA-C could induce glucose uptake in subcutaneous WAT through GLUT4, and we observed that ABA-C increased GLUT4 expression in the ABA-C-treated group compared with the control group (Figure 2C). In addition, ABA-C treatment stimulates brown adipocyte-specific gene UCP1 upregulation, in both subcutaneous WAT and BAT, to produce metabolic benefits (Figure 2D). However, we observed no significant difference in PGC1-α expression level, but only a moderate downregulation in BAT of ABA-C-treated vs. the control group (Figure 2E), suggesting a non-synergistic effect with UCP1 levels in the BAT. The little to no effect of PGC1-α in BAT may not be related to browning, since the WAT is the primary target tissue for ABA to promote WAT browning to increase energy metabolism. Collectively, these data demonstrate that ABA-C induces browning features in subcutaneous WAT of chronically treated mice, as indicated by increased mRNA levels of the brown adipocyte marker UCP1 and also adipogenesis-related genes, as well as apparent browning morphology.

### 3.4. Chronic ABA-C Treatment Induces Browning of Visceral WAT

**Figure 3:**
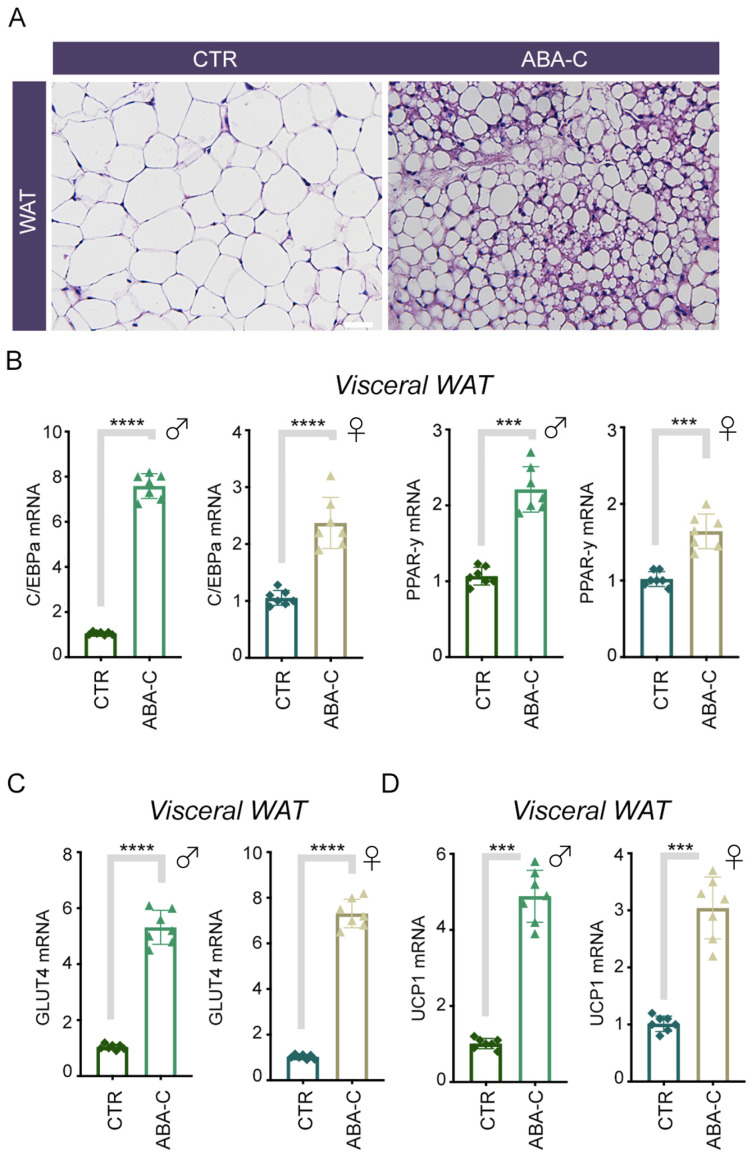
Browning of visceral WAT was induced by chronic ABA-C treatment. (**A**) Hematoxylin & eosin (H&E) staining was performed on WAT sections of ABA-C-treated and control (CTR) mice. Magnification 20×, scale bar 50 μm. (**B**–**D**) mRNA expression analysis of C/EBP-αEBPαa and PPAR-γ, GLUT4, and UCP1 in visceral WAT of ABA-C-treated animals compared with controls, in both males and females. Cyclophilin A was used as internal control. Normalized copies of the indicated genes in CTR were set as 1. The data represent the means ± SD from three separate experiments. *n* = 7. *** *p* < 0.001; **** *p* < 0.0001.

To evaluate the effect of ABA-C chronic treatment on visceral adipose tissue as well as subcutaneous adipose tissue, after 30 days of ABA-C treatment, we observed that histologically, ABA-C treatment induced browning of visceral WAT compared with untreated controls (Figure 3A). For this purpose, we measured the expression of C/EBP-αEBPαa and PPAR-γ in visceral WAT from ABA-C-treated and control groups. Again, the examined adipogenesis markers were upregulated in visceral WAT of ABA-C-treated animals compared with controls, both in males and females (Figure 3B). Moreover, we also investigated whether ABA-C could be an inducer of glucose uptake in visceral WAT via GLUT4, and we observed that ABA-C enhanced GLUT4 expression in the ABA-C-treated group compared with the control group (Figure 3C). Finally, ABA-C treatment upregulated the brown adipocyte-specific gene UCP1 in visceral WAT (Figure 3D). Taken together, these data suggest that chronic treatment with ABA induces the characteristics of browning even in visceral WAT.

### 3.5. ABA-C Improves Lipidemia and Reduces Adipose Tissue Weight

**Figure 4:**
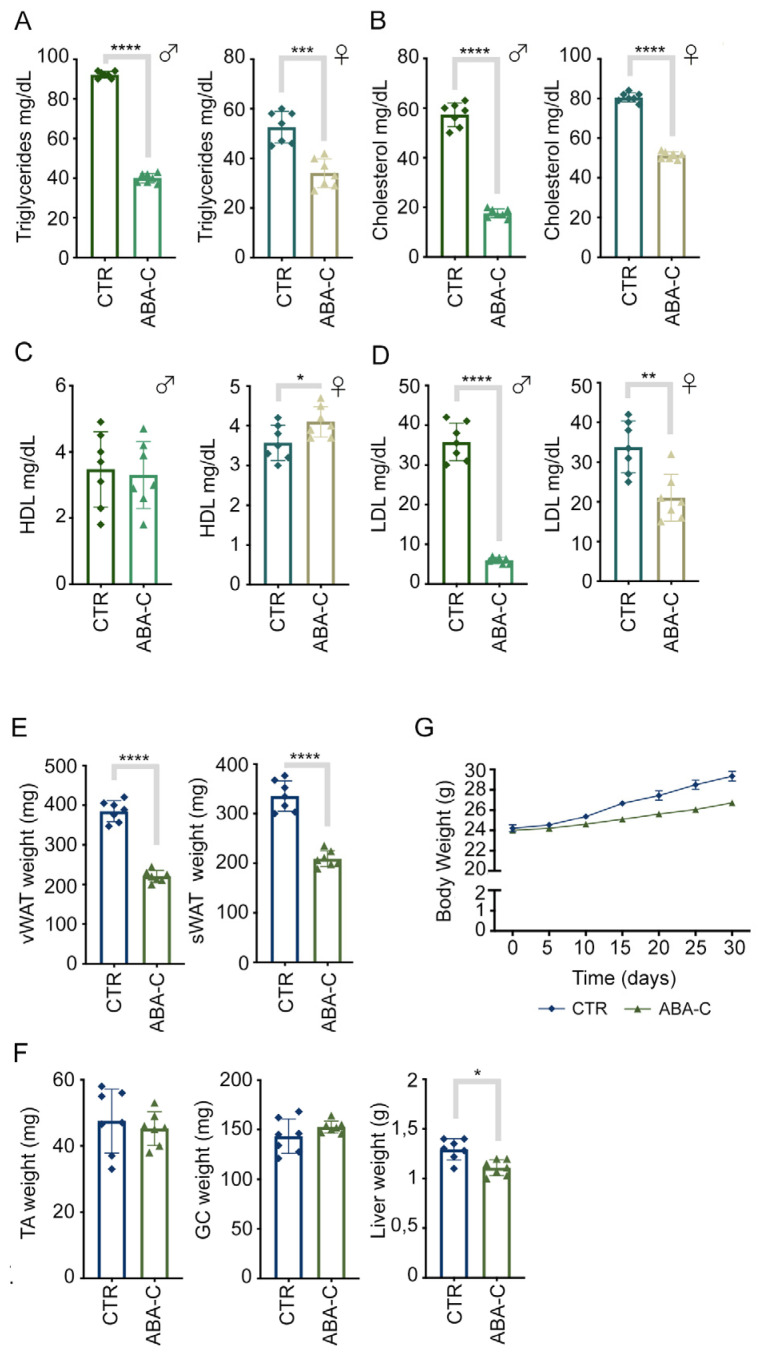
Chronic ABA treatment improves lipidemia. Analysis of lipid parameters (**A**–**D**) showed that chronic administration of ABA-C significantly reduced triglycerides, total cholesterol, and LDL cholesterol levels compared with controls. Analysis of (**E**,**F**) fat and lean mass (*n* = 7 per group) showed that mice treated with ABA-C had a reduction in fat mass but no change in lean mass. ABA-C-treated mice did not significantly lose weight compared with controls (**G**). The data represent the means ± SD from three separate experiments. *n* = 7. * *p* < 0.1; ** *p* < 0.01; *** *p* < 0.001; **** *p* < 0.0001.

The results of the herein study demonstrated that chronic administration of ABA-C significantly improved metabolic parameters. Indeed, blood chemistry parameters (Figure 4A–D), i.e., triglycerides, total cholesterol, and LDL cholesterol levels, were reduced in ABA-C-treated mice compared with controls, with a significant increase in HDL cholesterol levels observed only in female ABA-C-treated mice. In addition, ABA-C-treated mice showed no significant reduction in body weight compared with controls (Figure 4G), and the amount of food and water consumed did not change significantly with ABA-C administration. Conversely, ABA-C reduced the weight of WAT (both visceral and subcutaneous) and BAT (Figure 4E) but did not alter the lean mass; in fact, we did not observe any variation in the weight of the anterior tibial muscle (TA) and the gastrocnemius (GC) (Figure 4F).

### 3.6. Improvement in Glycemic Control with Acute ABA Treatment

**Figure 5:**
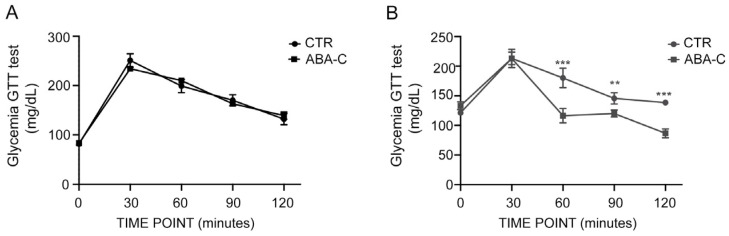
Acute ABA treatment improves glycemic control. Evaluation of glucose tolerance tests in both chronically (**A**) and acutely (**B**) ABA-treated mice compared with controls. Blood glucose levels were measured at baseline (0 min) and at 30, 60, 90, and 120 min following an intraperitoneal injection of beta D-glucose. The data represent the means ± SD from three separate experiments. *n* = 5. ** *p* < 0.01; *** *p* < 0.001.

To evaluate the effect of chronic ABA-C treatment on glycemic response, a glucose tolerance test (GTT) was performed on day 30 using 2 mg glucose/Kg BW, with glycemia monitored over 120 min. Unexpectedly, the glycemic profile after glucose challenge was not altered in ABA-treated mice compared with controls at any time point (Figure 5A). Based on previous results and the described effects on glycemic, chronic treatment does not show an improved response to glucose load, probably because the effect is lost in this timing. This led us to explore whether acute ABA-C treatment would improve glucose tolerance. A significantly reduced elevation of glycemia after glucose load was observed in ABA-treated mice compared with control mice (Figure 5B).

## 4. Discussion

An excess of WAT in humans, especially visceral adipose tissue (VAT), poses significant health risks as it contributes to the release of inflammatory markers and hormones that can impair organ function, leading to increased risks of cardiovascular diseases and metabolic disorders [24]. Notably, visceral WAT is more strongly associated with insulin resistance and metabolic disorders, while subcutaneous WAT generally has a different metabolic profile and is considered less detrimental in this context. Nevertheless, existing literature suggests that subcutaneous WAT is more responsive to browning stimuli than visceral WAT, which is often more resistant [6,7,8,9]. By analyzing the effects of ABA-C on each tissue type separately, we aimed to determine whether ABA-C would differentially promote browning and mitochondrial activity, potentially leading to specific metabolic benefits based on tissue type. The results of the present study demonstrate that chronic treatment with ABA-C induces mitochondrial biogenesis and enhances mitochondrial function in WAT, overall contributing to a browning effect. In this regard, the observed increase in mtDNA content and mitochondrial activity, as evidenced by MitoTracker™ staining, indicated a robust enhancement of mitochondrial function (Figure 1C,D). This result was further supported by the significant increase in the expression of UCP1 in both WAT and BAT of ABA-C-treated mice compared with the control group (Figure 2D–E and Figure 3D). The regulation of UCP1 expression is controlled by multiple transcriptional factors. Among them, PPAR-γ and C/EBPα play pivotal roles in initiating both white and brown adipogenesis [10,25], while PCG1-α and PDRM16 are crucial for brown fat determination, as they promote the expression of genes specific to brown adipose tissue [26,27]. Notably, an upregulation of PGC1-α and C/EBPα expression has been observed in adipocytes of the ABA-C-treated groups (Figure 1B, Figure 2B, and Figure 3B, respectively). As expected, UCP1 levels increased in the BAT of ABA-C-treated mice, but we did not see an increase in PGC1-α levels following treatment with ABA-C, probably because this latter has a primary effect on white adipose tissue (2E). In addition, histological analysis of subcutaneous and visceral WAT of treated mice revealed a marked increase in brown cell formation, characterized by smaller, more mitochondria-rich adipocytes (Figure 2A and Figure 3A). The reduction in adipocyte size and triglyceride content with ABA-C treatment further supports its role in adipose tissue remodeling. Unlike insulin and TZDs, which promote lipid accumulation [28,29], treatment with ABA-C is suggested to limit triglyceride storage, potentially offering a safer alternative for long-term metabolic regulation. The induction of mitochondrial activity is a critical aspect of adipose tissue browning, a process wherein white adipocytes gain characteristics of brown adipocytes, including high mitochondrial content and thermogenic capacity [30]. In this context, the findings that ABA-C-treated mice exhibit increased expression of browning markers such as UCP1 and PGC-1α underscore the potential of this supplementation to stimulate energy expenditure and combat obesity. Indeed, BAT plays a crucial role in regulating glucose homeostasis and enhancing insulin sensitivity [8], whereas brown adipogenesis is notably suppressed in individuals with insulin resistance [31]. Therefore, the transition of white adipocytes to a brown-like state, often referred to as “browning”, is a favorable strategy for enhancing metabolic health [32].

Another key finding of this study is the effect of ABA-C in enhancing GLUT4 expression in subcutaneous and visceral WAT (Figure 2C and Figure 3C). Previous research has established that ABA, through its receptor LANCL2, promotes the translocation of GLUT4 to the plasma membrane, enhancing glucose uptake in adipocytes [5]. This study corroborates these findings, demonstrating that ABA-C treatment led to significant upregulation of GLUT4 expression. As GLUT4 represents the primary glucose transporter in adipose tissue and its activity is essential for maintaining glucose homeostasis [33], the obtained data suggest that ABA-C treatment can enhance insulin sensitivity and improve glycemic control. This is particularly significant in the context of metabolic disorders such as diabetes, where impaired GLUT4 function is a hallmark. Indeed, the specific ablation of GLUT4 from adipocytes leads to systemic insulin resistance, highlighting its crucial role in glucose metabolism [34]. Further confirmation of the abovementioned result derives from the evidence that GLUT4 expression is significantly regulated by PPAR-γ, which is activated by thiazolidinediones (TZDs) to mediate antidiabetic effects [30,35]. Interestingly, our study demonstrates the ability of ABA-C treatment to enhance the expression of PPAR-γ in WAT (Figure 2B and Figure 3B). Typically, upregulation of PPAR-γ alone can suppress GLUT4 transcription unless PPAR-γ is bound to coactivators or ligands (e.g., TZDs), which causes it to disassociate from the GLUT4 promoter [36]. PGC1α, when overexpressed, binds to PPAR-γ, thereby increasing GLUT4 expression and facilitating glucose transport in muscle cells [37]. Therefore, the simultaneous increase in PGC1-α transcription induced by ABA-C (Figure 1B) might be responsible for the upregulation in GLUT4 expression.

Interestingly, the results from Section 3.5 demonstrate that chronic supplementation with ABA-C significantly improves lipid metabolism and reduces adipose tissue mass. Specifically, the reduction in triglycerides, total cholesterol, and LDL cholesterol aligns with the effects observed in the study by Magnone et al. (2018), where low-dose ABA improved glycemic and lipid profiles in both human and murine models [38]. The mechanisms underlying these improvements are likely linked to the upregulation of adipose-specific markers and mitochondrial activity, as evidenced by increased UCP1 expression and mitochondrial DNA, and the insulin-sparing effect of ABA. By reducing the reliance on insulin for glucose metabolism, ABA minimizes chronic insulin secretion, which is often associated with enhanced lipogenesis and triglyceride storage [29]. Overall, the improvements in lipid metabolism, coupled with the observed reductions in visceral and subcutaneous WAT, carry profound implications for metabolic health and highlight the ABA dual role in enhancing energy expenditure and improving lipid homeostasis. To our knowledge, this is the first study to directly evaluate the weight of adipose tissue in response to ABA supplementation. Unlike previous investigations that focused primarily on markers of metabolic improvement, the present study provides a quantitative assessment of visceral and subcutaneous WAT, as well as BAT (Figure 4E), providing direct evidence of its impact on adipose tissue remodeling. Another significant observation is the lack of substantial changes in overall body weight despite the reductions in adipose tissue. This suggests that ABA-C exerts its effects through direct metabolic modulation rather than by inducing calorie deficits or altering feeding and drinking behaviors.

As regards the effects on glycemic control, data of the herein study demonstrate that while chronic ABA-C treatment did not significantly alter the glycemic profile in GTT performed at 30 days (Figure 5A), acute treatment showed a notable improvement in glucose tolerance (Figure 5B). This suggests that the timing and mode of administration with nutraceutical supplementations based on ABA are critical factors in its metabolic effects, as the different outcomes in the glycemic profile between acute and chronic ABA-C treatments likely derive from the distinct physiological mechanisms activated by these two modes of administration. Acute ABA-C treatment seems to exert an immediate effect on glycemic control, possibly through rapid stimulation of glucose uptake pathways and a swift increase in GLUT4 translocation in adipose tissues, enhancing glucose disposal shortly after administration. In contrast, chronic ABA-C treatment may lead to longer-term adaptations, primarily influencing mitochondrial biogenesis, adipose tissue browning, and metabolic remodeling. These processes, while beneficial for overall metabolic health, may not produce an immediate impact on glycemic levels in the glucose tolerance test. Thus, the acute treatment provides a short-term enhancement in glucose uptake, whereas chronic treatment appears to contribute to gradual metabolic improvements that could lead to sustained insulin sensitivity and energy expenditure rather than immediate glycemic effects. Based on the results presented in the herein study, supplementation with ABA-C as a source of ABA arises as a useful tool to manage and mitigate VAT and its associated risks. Overall, the finding that oral ABA-C supplementation in rodents promotes WAT to acquire brown features by WAT while enhancing glucose uptake and increasing UCP1 levels in BAT presents a promising basis for further in vivo studies on BAT function. Additionally, current data, along with previously published evidence [2,14], strongly support the hypothesis that ABA and insulin have distinct roles in adipose tissue. It is well recognized that oral glucose load increases plasma levels of both ABA and insulin [39], with two different biological effects: insulin mainly targets WAT, promoting glucose entrance, pre-adipocyte differentiation, and triglyceride accumulation [29], while ABA, on the contrary, stimulates glucose uptake by BAT, preserves the release of insulin, and induces the expression of browning genes and mitochondrial biogenesis in WAT [14]. Through the insulin-sparing effect, this hormone reduces the impact of insulin on WAT. Consequently, the external supplementation with ABA from vegetable sources may positively regulate body weight homeostasis and the WAT-to-BAT ratio, making it an attractive nutraceutical treatment to improve the dysmetabolic conditions related to obesity and insulin resistance.

## 5. Conclusions

In conclusion, this study provides robust evidence that ABA-C supplementation modulates glucose metabolism and adipose tissue function through multiple mechanisms. The induction of GLUT4 expression, mitochondrial biogenesis, and adipose tissue browning highlights its potential as a therapeutic agent for managing obesity, diabetes, and correlated metabolic disorders. Moreover, the ability of ABA contained in ABA-C to improve glucose uptake without increasing triglyceride accumulation distinguishes it from other agents like insulin and TZDs, which often promote lipid storage. Future research should explore the clinical applications of infusions based on thinned nectarines in human subjects, focusing on their efficacy, optimal dosing, and long-term safety. The promising results from animal models suggest that ABA-C supplementation could be a valuable tool in the fight against metabolic diseases, especially impaired glucose homeostasis, offering a natural and effective means of enhancing metabolic health.

## Data Availability

The original contributions presented in the study are included in the article/Supplementary Materials, further inquiries can be directed to the corresponding author.

## Supplementary Materials

The following supporting information can be downloaded at https://www.mdpi.com/article/10.3390/foods14010048/s1, Table S1: Oligonucleotides used for real-time PCR and mtDNA copy number analysis.
