# Supplementary material for: AbaComplex Enhances Mitochondrial Biogenesis and Adipose Tissue Browning: Implications for Obesity and Glucose Regulation
Source: Foods. 2024 Dec 27;14(1):48. doi: 10.3390/foods14010048 (PMC11720057; doi:10.3390/foods14010048)
Supplement: Supplementary file 1 [file foods-14-00048-s001.zip › foods-3269158-supplementary.pdf]

Table S1. Oligonucleotides used for real-time PCR.

Gene	**Forward primer (5' → 3')**	**Reverse primer (5' → 3')**
CypA	CGCCACTGTCGCTTTTCG	AACTTTGTCTGCAAACAGCTC
*P*gc*1-α*	TGATGTGAATGACTTGGATACAGACA	GCTCATTGTTGTACTGGTTGGATATG
C/ebpa	GTCGGTGGACAAGAACAGCA	CCTTCTGTTGCGTCTCCACG
Ppar-γ	GGAAGACCACTCGCATTCCTT	GTAATCAGCAACCATTGGGTCA
*U*cp*1*	GTCGGTCCTTCCTTGGTGTA	GGGCCCTTGTAAACAACAAA
*G*lut*4*	CTGTGCCATCCTGATGACTG	GGGTTTCACCTCCTGCTCTA
		Oligonucleotides used for mtDNA copy number analysis
*N*d*1*	AACACTCCTCGTCCCCATTC	GTGAGTGATAGGGTAGGTGCA
RNAseP	GCCTACACTGGAGTCGTGCTACT	CTGACCACACGAGCTGGTAGAA
